# Mitochondrial Genomes of the American Dog Tick (Dermacentor variabilis) Isolated from Horses in the Midwestern United States

**DOI:** 10.1128/mra.00330-22

**Published:** 2022-12-21

**Authors:** Samantha Reynolds, Makaela Hedberg, Brian Herrin, Jeba R. J. Jesudoss Chelladurai

**Affiliations:** a Department of Diagnostic Medicine/Pathobiology, Kansas State University College of Veterinary Medicine, Manhattan, Kansas, USA; University of California, Riverside

## Abstract

Here, we report two complete and three partial mitochondrial genome sequences of Dermacentor variabilis specimens collected from horses in the United States. The complete genomes are 14,837 bp long and contain 13 protein-coding genes, 2 rRNA genes, and 22 tRNA genes. The sequences have been deposited under GenBank accession numbers ON052120 to ON052124.

## ANNOUNCEMENT

Dermacentor variabilis (Say, 1821) is an important ectoparasite that is capable of parasitizing a wide variety of mammals, including humans. It is distributed discontinuously from southern Canada to the Gulf Coast of Mexico and can expand further due to climate change (1, 2). It is an important vector of Rickettsia rickettsii (3), other *Rickettsia* species (4), Francisella tularensis (5) and is an experimental vector of Cytauxzoon felis (6). Despite its importance, complete mitochondrial genomes of only two isolates, from Georgia, USA (GenBank accession number MN165636.1), and Oklahoma, USA (GenBank accession number MN175686.1), have been sequenced. To contribute to larger phylogeographic efforts, we present two complete mitochondrial genome sequences of Dermacentor variabilis specimens isolated from horses in Wisconsin, USA, and three partial mitochondrial genome sequences of specimens from Kansas and Illinois, USA.

Dermacentor variabilis adults were isolated from the pelage of horses as part of the National Equine Tick Survey (7) and identified using established keys (8). Exoskeletons are retained as voucher specimens (accession numbers DvarWI152-3, DvarWI152-4, DvarIL245-1, DvarIL245-2, and DvarKSKon15) at the Kansas State University College of Veterinary Medicine. Genomic DNA was extracted from the specimens using the DNeasy blood and tissue kit (Qiagen). Complete mitochondrial genomes were amplified as two overlapping fragments, L3 and L4, using primers GCTAKTGGGTTCATACCCCAA, CGACCTCGATGTTGGATTAGGA, CCAACCTGATTCWCATCGGTCT, and TCATCGCGGTAAAATGACTGA (annealing temperature 55°C) as described previously (9). Amplicons were fragmented, sequencing libraries were prepared using the NEBNext Ultra DNA library preparation kit (New England Biolabs), and libraries were pooled and sequenced on an Illumina MiSeq instrument in paired-end 150-bp read mode. An average of 100,710 paired-end reads were generated for each sample. Sequences were analyzed with FastQC v0.73 (10), adapters were trimmed using Trimmomatic v0.38.1 (11), and sequences were mapped to the reference genome (GenBank accession number NC_061217.1) using Minimap2 v2.24 (12). Consensus sequences were extracted using the Integrative Genomics Viewer (IGV) v2.12.3 (13) based on parameters described previously (14). The average coverage was 577× for the complete mitochondrial sequences. Annotation was performed using Mitos v2 (15).

The complete mitochondrial genomes are 14,837 bp and contain 13 protein-coding genes, 22 tRNA genes, and 2 control regions. Nine genes (*nad2*, *cox1*, *cox2*, *atp8*, *atp6*, *cox3*, *nad3*, *nad6*, and *cytb*) are encoded on the positive strand and four (*nad1*, *nad5*, *nad4*, and *nad4L*) on the negative strand. The GC content is low at 21.3%, typical of metastriate mitochondrial genomes (16). Comparison of the complete mitochondrial genomes revealed similarity of 99.18% between the Wisconsin isolate and the reference Georgia isolate (GenBank accession number NC_061217.1).

**FIG 1:**
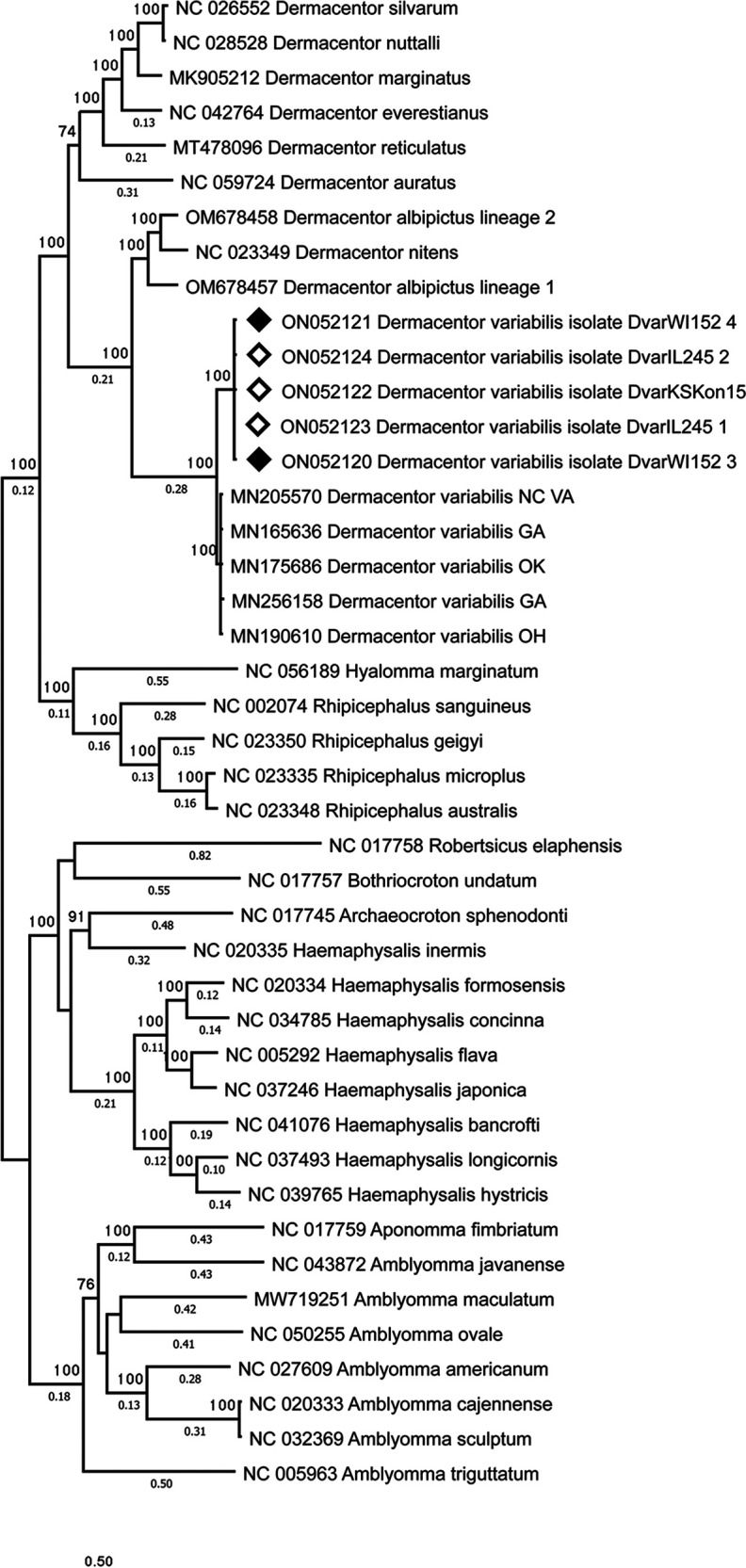
Maximum likelihood phylogenetic tree of 15 mitochondrial genes (13 protein-coding genes and 2 rRNA genes) constructed with the GTR+G+I model in PhyML to infer the relationship of Dermacentor variabilis to other selected tick species in the family Ixodidae (GenBank accession numbers precede biological names). There were 11,746 bp in the final data set. Complete mitochondrial genomes from this study are marked with black diamonds and partial mitochondrial genomes with white diamonds.

Sequences for 13 protein-coding genes and 2 rRNA genes obtained in this study and a selected set of tick sequences from GenBank were compiled. Individual gene sequences were aligned with MAFFT (17), trimmed with trimAI (18), and concatenated in the same order as in the genome. A 15-gene maximum likelihood phylogenetic tree was created using PhyML-SMS (19, 20) and included 11,746 bp of concatenated nucleotides (Fig. 1). Dermacentor variabilis forms a monophyletic clade with a high level of support.

### Data availability.

The mitochondrial genome sequences from the study have been deposited in GenBank under the accession numbers ON052120 to ON052124. Raw reads have been deposited under NCBI BioProject accession number PRJNA885479 and SRA accession numbers SRX17751554 to SRX17751558.
